# Idiopathic left ventricular tachycardia following atrioventricular conduction block by rapid atrial tachycardia

**DOI:** 10.1002/ccr3.940

**Published:** 2017-06-01

**Authors:** Kazuyoshi Suenari, Naoya Mitsuba, Hidekazu Hirao, Hironori Ueda, Yasuki Kihara

**Affiliations:** ^1^ Department of Cardiovascular MedicineHiroshima University Graduate School of Biomedical SciencesHiroshimaJapan; ^2^ Department of CardiologyHiroshima Prefectural HospitalHiroshimaJapan

**Keywords:** Atrial tachycardia, catheter ablation, tachycardia‐mediated cardiomyopathy, ventricular tachycardia

## Abstract

The present case demonstrated a rare situation alternating between a repetitive atrial tachycardia (AT) and ventricular tachycardia (VT). A unique induction mechanism was noted in which the VT was induced after Wenckebach AV node conduction block following the repetitive rapid AT.

## Case Report

**Figure 1:**
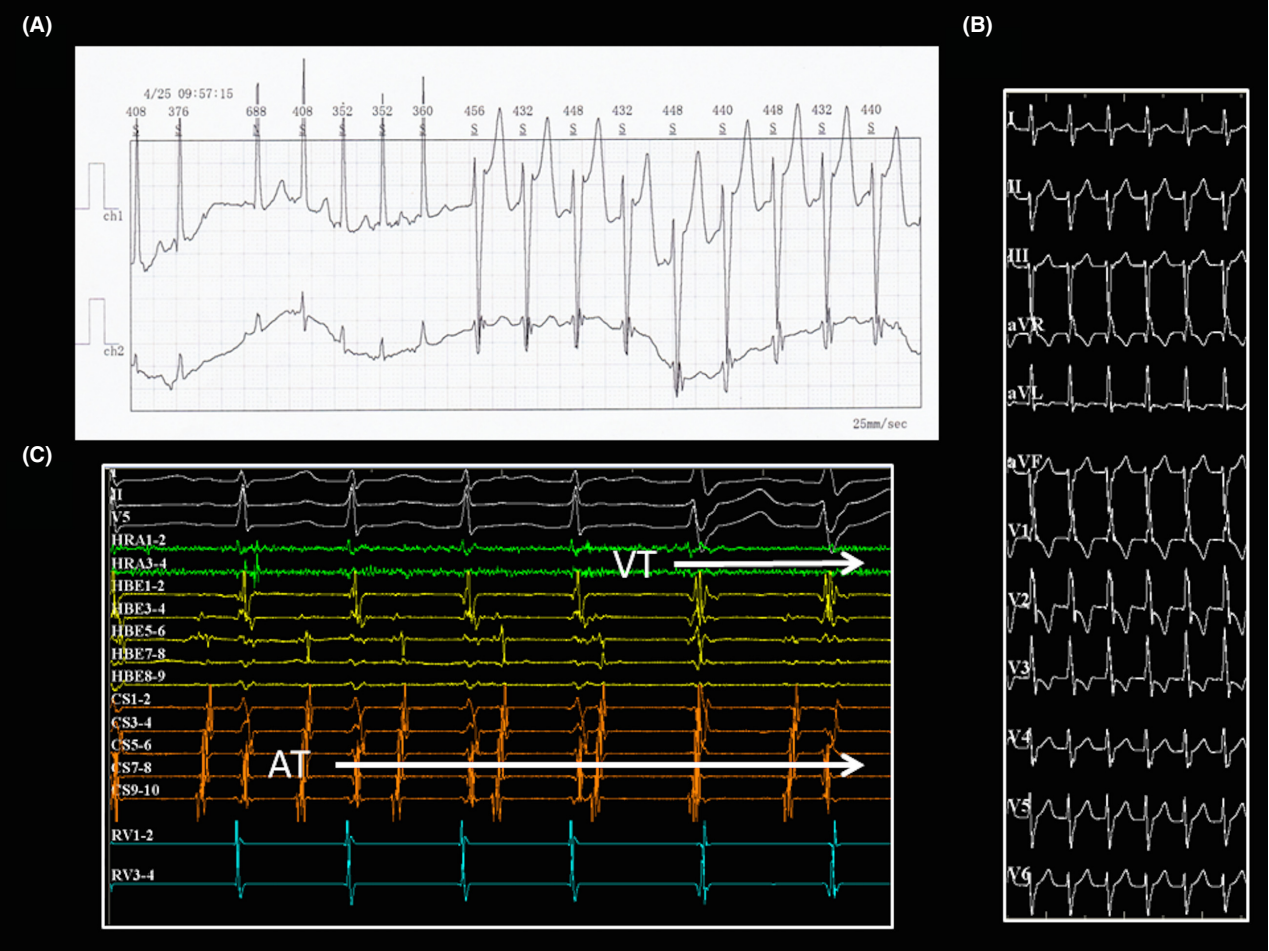
(A) A 24‐hour Holter ECG recording. Almost all of his tachycardia episodes were a repetitive narrow QRS tachycardia (total heart beats; 185,715 beats, average heart rate; 134 beats per min) and sometimes changed from a narrow to wide QRS complex (12% of the total amount of heart beats and a maximum of 58 sustained beats) during the recording. (B) A 12‐lead ECG during the ILVT. (C) The ILVT was spontaneously induced following Wenckebach AV node conduction block during the repetitive AT. The tachycardia cycle lengths of the AT and VT were 385 and 487 msec, respectively. AT, atrial tachycardia; ILVT, idiopathic left ventricular tachycardia.

**Figure 2:**
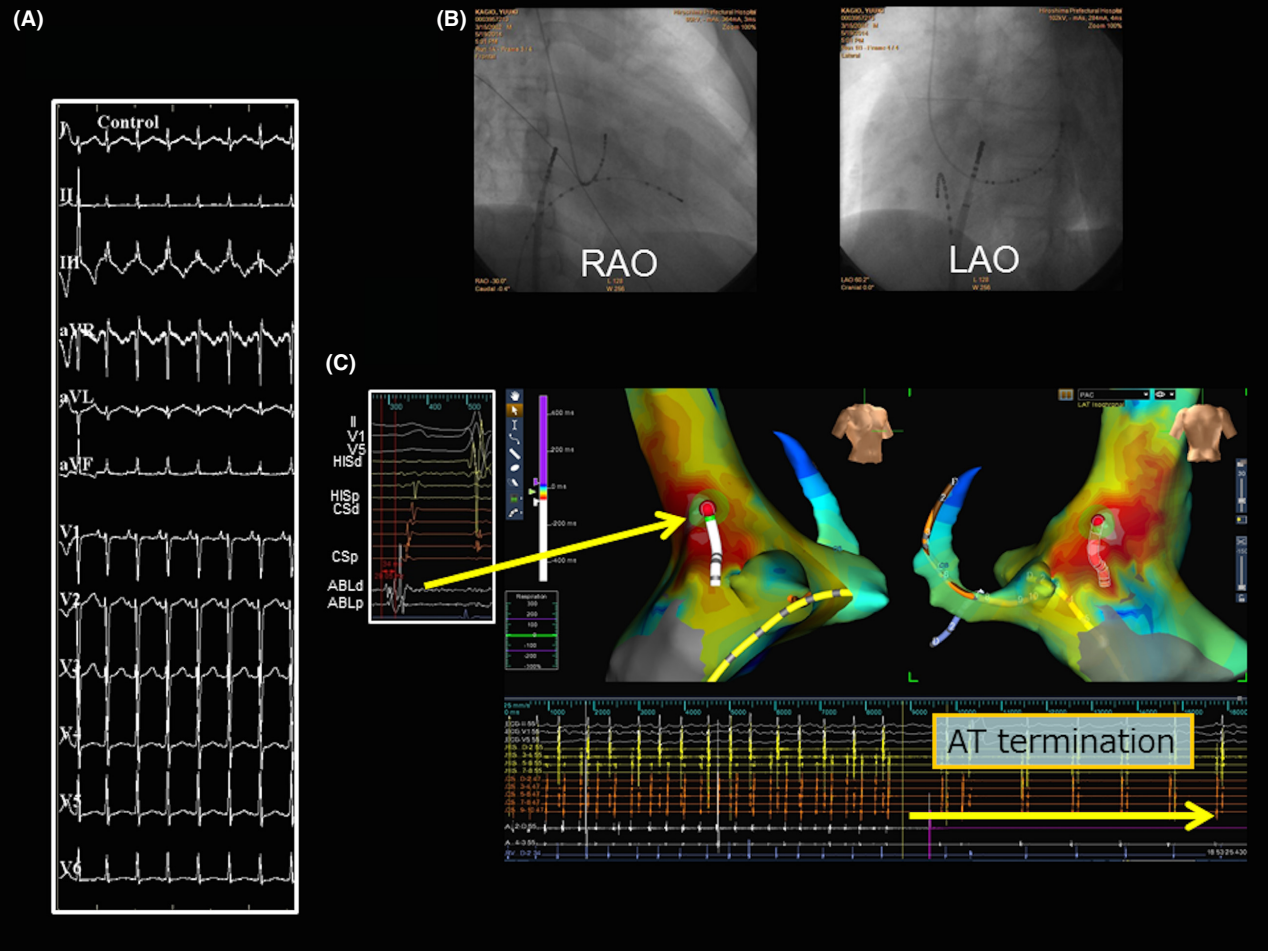
Activation mapping with Ensite Velocity and the fluoroscopic view of the successful ablation site of the repetitive AT. (A) A 12‐lead ECG during the AT. (B) Fluoroscopic images (RAO and LAO views) showing the successful ablation site. (C) The activation map and successful ablation site including the local atrial electrograms recorded by the ablation catheter are demonstrated with Ensite Velocity. Intracardiac recordings show that successful AT termination was achieved during the ablation at that site. RAO, right anterior oblique; LAO, left anterior oblique.

**Figure 3:**
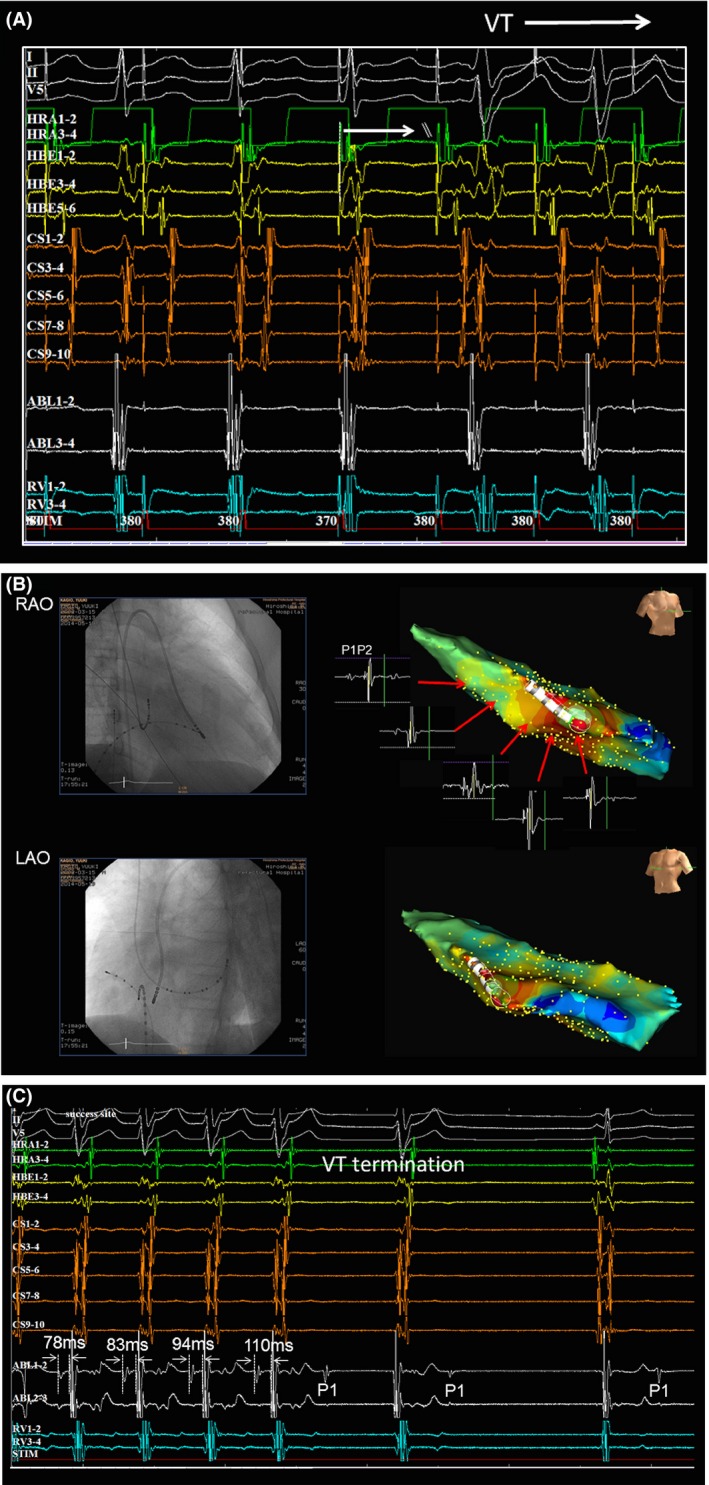
(A) Burst pacing from the HRA with a similar cycle length as the clinical AT (pacing cycle length: 380 msec) gradually prolonged the AV interval and induced wide QRS tachycardia following Wenckebach AV node conduction block. (B) Activation mapping and fluoroscopic view of the successful ablation site of the ILVT. During VT, P1 and P2 were recorded along the left posterior fascicle region from the distal tip of ablation catheter. (C) During the energy application, the P1‐QRS interval gradually prolonged, and the ILVT was terminated by block between P1 and the QRS. The P1 occurred after the QRS complex during sinus rhythm.

A narrow QRS tachycardia without any symptoms was occasionally detected by 12‐lead ECGs during school medical checkups in a 12‐year‐old boy. The baseline ECG recording in our outpatient clinic demonstrated a repetitive, narrow‐complex, long RP tachycardia with a 1:1 A‐V relationship during a rate of 130 beats per min (bpm). Almost all of his tachycardias were a repetitive narrow QRS tachycardia and sometimes changed from a narrow to wide QRS complex during a Holter ECG recording (Fig. 1A). Furthermore, his echocardiogram revealed severe and diffuse LV dysfunction (LV ejection fraction: 34%) and heart failure (NT‐proBNP: 1068 pg/mL). At the beginning of the electrophysiological study, a wide QRS tachycardia with AV dissociation was spontaneously induced following Wenckebach AV node conduction during the repetitive atrial tachycardia (AT) (Fig. 1B and C). The 12‐lead ECG of this wide QRS tachycardia presented with an RBBB morphology and northwest axis deviation (Fig. 1B). As most of his tachycardias were almost of an incessant AT nature during the procedure (Fig. 2A), we first started to map it. Based on the activation mapping of the AT with the EnSite Velocity system (St. Jude Medical, Minnetonka, MN), we successfully ablated the earliest activation site at the right atrial posteroseptum (34 msec earlier than the onset of the surface P wave, Figure 2B and C). After the ablation, his AT was no longer inducible. Burst pacing from the high right atrium with a similar cycle length as the clinical AT (pacing cycle length: 380 msec) gradually prolonged the AV interval and induced the wide QRS tachycardia following Wenckebach AV node conduction block (Fig. 3A). The local ventricular electrograms of the left septal region recorded a diastolic potential (P1), presystolic Purkinje potential (P2) preceding the ventricular activation, and His bundle potential following the onset of the QRS complex during the ventricular tachycardia (VT) (Fig. 3B). After obtaining an activation map of the P2 potential using EnSite Velocity, we ablated the mid‐septal site of the left ventricle, where the earliest P2 potential was recorded (Fig. 3B‐right side). During the energy application, the P1‐QRS interval gradually prolonged, and the idiopathic left VT (ILVT) was terminated by block between P1 and the QRS (Fig. 3C). After the successful ablation, the P1 occurred after the QRS complex during sinus rhythm (Fig. 3C). Finally, atrial programmed stimulation and ventricular programmed stimulation were not able to induce either the AT or ILVT under an isoproterenol infusion. None of the arrhythmias in this case recurred during a 1‐year follow‐up. Although his LV diastolic diameter did not change much (55.8–55.5 mm), his LV dysfunction and NT‐pro BNP recovered (LV ejection fraction: 61%, NT‐proBNP: 69 pg/mL). Moreover, no change in the QRS morphology was observed after the ablation.

## Discussion

Idiopathic left VT was previously reported to be sensitive to verapamil and to have an RBBB morphology with a left axis configuration on the electrocardiogram. For left posterior fascicular VT, the reentrant VT circuit involving the left posterior fascicle is considered to consist of specialized Purkinje tissue with decremental properties and verapamil sensitivity, is located near the distal third of the left posterior fascicle Purkinje network (diastolic potential: P1), and serves as the anterograde limb, and the left posterior fascicle or Purkinje fibers near the left posterior fascicle with faster conduction, that is, normal fibers (presystolic Purkinje potential: P2), serve as the retrograde limb. There could be a link in the ventricular myocardium that serves as a bridge between P1 and P2. Therefore, the Purkinje potentials indicate the successful ablation target site 1. This type of VT is also well known to be inducible by atrial programmed stimulation. Although the ILVT in the present case was not inducible by programmed stimulation from the right ventricle electrode catheter, the ILVT was inducible during burst pacing from the high right atrium (HRA) only following AV node conduction block as with the spontaneous induction. According to the intracardiac recording (Fig. 3A), the AV node conduction gradually prolonged by HRA burst pacing and the ILVT was initiated after AV node conduction block. Moreover, the tachycardia cycle length of his ILVT (tachycardia cycle length [TCL] = 487 msec) was much slower than that of the repetitive AT (TCL = 385 msec). Thus, the induction method of his ILVT may have reflected the establishment of reentry by creating slow conduction in an abnormal part of the specialized conduction system (i.e., the Purkinje fiber network of the left posterior fascicle) as a decelerating ventricular response due to the rapid AT. This could be a reason for a less frequent occurrence of ILVT in clinical practice. Several previous investigators reported that VT is able to be induced by atrial stimulation 2, 3, 4, 5. Most of those reports demonstrated that a single conducted atrial premature stimulus after high rate pacing could be the only requirement to create unidirectional block within the reentrant circuit near the left posterior fascicle without interruption of the normal intraventricular conduction system. Zipes et al. reported that VT initiation by atrial stimuli, when similar premature ventricular stimuli failed to induce the VT, indicated a dependence on the geometry and timing of the premature wavefront 4. On the other hand, Berman et al. reported that the VT was initiated by both normally and aberrantly conducted atrial premature beats 6. Therefore, the present case would have suffered from ILVT attacks induced by spontaneous PACs if we had treated only his AT. Finally, we successfully treated both tachyarrhythmias and his LV dysfunction recovered during the follow‐up period.

In summary, we described a tachycardia‐mediated cardiomyopathy case with an anterogradely induced ILVT following AV node conduction block by a rapid AT.

## Authorship

KS, NM, and HH: were involved in the clinical care of the patient. KS: did the literature review and drafted the manuscript. HU and YK: critically reviewed the manuscript for important intellectual content.

## Conflict of Interest

The authors have no conflict of interest or funding to disclose.
